# Sja-Let-7 Attenuates Carbon Tetrachloride-Induced Liver Fibrosis in a Mouse Model via Col1α2

**DOI:** 10.3390/biology12121465

**Published:** 2023-11-24

**Authors:** Haoran Zhong, Bowen Dong, Danlin Zhu, Hao Li, Ke Lu, Zhiqiang Fu, Jinming Liu, Yamei Jin

**Affiliations:** 1 National Reference Laboratory for Animal Schistosomiasis, Shanghai Veterinary Research Institute, Chinese Academy of Agricultural Sciences, Shanghai 200241, China; leslie1996716@163.com (H.Z.); dongbw131420@163.com (B.D.); 13957236028@163.com (D.Z.); lihao@shvri.ac.cn (H.L.); luke@shvri.ac.cn (K.L.); fuzhiqiang@shvri.ac.cn (Z.F.); jimyliu@shvri.ac.cn (J.L.); 2 Key Laboratory of Animal Parasitology of Ministry of Agriculture and Rural Affairs, Shanghai Veterinary Research Institute, Chinese Academy of Agricultural Sciences, Shanghai 200241, China

**Keywords:** liver fibrosis, hepatic stellate cell, sja-let-7, Col1α2, TGF-β/Smad signaling pathway

## Abstract

**Simple Summary:**

Liver fibrosis (LF) is a common pathologic feature of multiple liver diseases. However, there is currently no effective treatment to slow the progression of LF. In this study, sja-let-7 from *Schistosoma japonicum* was shown to regulate host immune responses and attenuate the progression of carbon tetrachloride-induced LF via the Col1α2 and TGF-β/Smad signaling pathway, thereby providing references for the application of worm-derived molecules for the treatment of LF.

**Abstract:**

Liver fibrosis (LF) is a chronic progressive disease with no definitive treatment. The aim of this study was to assess helminth-derived molecules as potential therapeutic targets to prevent or reverse LF. A mouse model of carbon tetrachloride (CCL_4_)-induced LF was established and sja-let-7 was overexpressed by treatment with a miRNA agomir once per week. After four weeks, serum biochemistry, hepatic hydroxyproline content measurements, liver histology, mRNA expression profiling of fibrotic markers, the dual-luciferase reporter assay, and fluorescence in situ hybridization (FISH) were performed. Administration of the sja-let-7 agomir markedly ameliorated hepatosplenomegaly and reduced the liver hydroxyproline content. Liver histological analysis showed significant reductions in collagen deposition in the sja-let-7 agomir-treated mice. Additionally, the mRNA levels of both pro-fibrotic markers and pro-inflammatory cytokines were diminished after treatment. Furthermore, the dual-luciferase reporter assay and FISH identified the α2 chain of collagen type 1 (Col1α2) as the direct target of sja-let-7. Accordingly, the progression of LF was attenuated by targeting Col1α2 and the TGF-β/Smad signaling pathway.

## 1. Introduction

Liver fibrosis (LF) is a common pathological feature of a variety of liver diseases and it is considered to be a wound-healing response to tissue injury, predominantly triggered by a combination of inflammatory and immune-mediated mechanisms [1]. However, there is currently no effective treatment. LF is a dynamic equilibrium involving interactions among multiple cell types, molecules, and signaling pathways, primarily involving hepatic stellate cells (HSCs), which store vitamin A in lipid droplets during quiescent conditions [2]. After stimulation, quiescent HSCs transdifferentiate into collagen-producing hepatic myofibroblast-like cells, which are characterized by the loss of lipid droplets and stores of vitamin A [3]. Upon proliferation and migration, HSCs produce excessive amounts of extracellular matrix proteins and cause an increased production of pro-inflammatory and pro-fibrogenic cytokines [1]. Simultaneously, HSCs increase the production of α-smooth muscle actin (α-SMA) and type Ⅰ collagen, which are established biomarkers of LF [4].

*Schistosoma japonicum* is a parasitic helminth that can produce offspring within a host for relatively prolonged periods [5]. Approximately half of schistosome eggs remain trapped within the host, accounting for the primary pathology of schistosomiasis, which is characterized by egg-induced granuloma formation and LF due to the host’s immune response [6]. Schistosomes employ intricate molecular mechanisms to modulate host immunity [7]. As schistosomes migrate within the host, the T helper (Th) type 1 response against worms in the early stage of acute infection switch to a strong Th2 response against the eggs trapped in tissues, which can be regulated by extracellular vesicles (EVs) derived from schistosome eggs [8]. A previous study [8] found that sja-miR-71a carried by schistosome egg-derived EVs acts as an anti-fibrotic factor and helps to maintain the balance of Th1/Th2/Th17 and regulatory T cells. Interestingly, the egg EV-derived miRNAs sja-miR-2162 [9] and a novel miRNA-33 [10] promote progression of LF via the TGF-β/Smad signaling pathway. These opposing effects demonstrate the dynamics of parasite–host interactions.

The terms “hygiene hypothesis” and “old friend hypothesis” have been applied to describe the unique immune regulatory mechanisms of helminths and interactions within hosts. Helminth-derived proteins, peptides, and miRNAs have generated growing interest as therapeutics for inflammatory diseases in humans, such as LF [11]. As the main component of soluble egg antigen, *S. japonicum* protein p40 was shown to inhibit the activation and proliferation of HSCs and decrease the expression of the α1 chain of collagen type 1 (Col1α1) [12]. Hence, *S. japonicum*-derived factors associated with the pathogenesis and progression of LF present potential therapeutic targets.

In this study, sja-let-7 derived from the EVs of *S. japonicum* worms was overexpressed in a mouse model of carbon tetrachloride (CCL_4_)-induced LF to determine whether targeting the α2 chain of collagen type 1 (Col1α2) and the TGF-β/Smad signaling pathway can attenuate the progression of LF.

## 2. Materials and Methods

### 2.1. Ethics Statement

All protocols involved in the animal experiments were performed according to the guidelines of the Committee for the Care and Use of Laboratory Animals of the Shanghai Veterinary Research Institute (Shanghai, China, permit no. SYXK-20160010) and were approved by the Ethics and Animal Welfare Committee of the Shanghai Veterinary Research Institute, Chinese Academy of Agricultural Sciences (Shanghai, China, experiment no. SV-20230505-03).

### 2.2. Animals

Specific-pathogen-free male BALB/c mice (6–8 weeks old; body weight 18 ± 2 g) were purchased from Shanghai Jiesijie Laboratory Animal Co., Ltd. (Shanghai, China) and were randomly divided into certain groups before the start of the study. Briefly, 12 BALB/c mice were intraperitoneally injected with CCL_4_ (0.1 mL 10% CCL_4_ diluted in peanut oil) (Sinopharm, Shanghai, China) twice a week for 4 weeks, which were divided into CCL_4_, CCL_4_+NC agomir, and CCL_4_+Sja-let-7 agomir groups (*n* = 4). In the CCL_4_+NC agomir and CCL_4_+Sja-let-7 agomir groups, mice were injected with 120 μL of 1 OD (optical density) NC or Sja-let-7 agomir (GenePharma, Shanghai, China) via the tail vein in the first week, and were intraperitoneally administrated once a week for the following three weeks, respectively (CCL_4_ administration on its own caused the mice’s body condition to decline, resulting in a reduced success rate for tail vein injections). In the control group, mice were intraperitoneally injected with 0.1 mL peanut oil twice a week for four weeks and were injected with 120 μL PBS once a week for four weeks (*n* = 4). Livers, spleen tissues, and blood samples from each mouse were harvested 24 h after the final CCL_4_ injection for further experiments. The detailed sequences of miRNA agomirs are shown in Table S1.

### 2.3. Liver and Spleen Index

Whole livers and spleens were harvested and measured. The extent of liver damage was evaluated macroscopically, noting any changes in liver color and stiffness. The livers and spleens were weighed and the indexes were calculated as the ratio of each respective organ to the animal’s body weight [13].

### 2.4. Hematological Analyses

Blood samples (~150 μL/mouse) were collected into K2 EDTA tubes (Solarbio, Beijing, China), and the complete blood count (CBC) assay was conducted on a Mindray BC-6800 Plus analyser (Mindary, Shenzhen, China). White blood cells (WBC) were divided into five categories: neutrophil (Neu), lymphocyte (Lym), monocyte (Mon), eosinophil (Eos), and basophil (Bas).

### 2.5. Liver Enzyme Quantification

For liver function assessment, the hydroxyproline, aminotransferase (ALT), and aspartate aminotransferase (AST) of the liver tissues were measured by commercial kits (Nanjing Jiancheng Bioengineering Institute, Nanjing, China) according to the manufacturer’s instructions.

### 2.6. Enzyme Linked Immunosorbent Assay (ELISA)

Blood samples (~250 μL/mouse) were collected and allowed to stand for 10 min, followed by centrifugation at 3000× *g* for 15 min at 4 °C. The serum in the supernatant was used to detect the α-SMA levels through a commercial mouse α-SMA ELISA kit (MLBio, Shanghai, China). The procedures were performed according to the manufacturer’s instructions.

### 2.7. Liver Histological, Immunohistochemistry and Immunofluorescence Analysis

The liver samples were fixed with 4% formaldehyde solution at room temperature (RT) for 48 h, dehydrated in ethanol, cleared in xylene, embedded in paraffin, and sliced into 5 µm sections for pathological observation using standard H&E staining (Servicebio, Wuhan, China). The extent of liver fibrosis was determined using Masson’s trichrome staining (Servicebio, Wuhan, China) and Sirius red staining (Servicebio, Wuhan, China). In the H&E staining slices, liver fibrosis and inflammation were assessed through the Ishak index score, which is a scoring system commonly used in liver fibrotic disease [14]. In the Masson and Sirius red staining slices, the percentage of positive staining area was also measured using ImageJ software Version 1.53t. Additionally, polarization microscopy (ZEISS, Oberkochen, Germany) was used to differentiate type I and type III collagen fibers and to quantitate their proportions in the Sirius red staining slices [15]. Type I collagen fibers were recognized by its thick fibers and bright red or yellow staining, while type III collagen fibers were characterized by slander fibers and green staining. The quantitative measurements of type I and type III collagen fibers were measured using ImageJ software.

Immunohistochemical assay (IHC) was performed using serial paraffin sections as described above, which were incubated with primary antibodies of α-SMA, Col1α1, Col1α2, Col3α1, TGF-β, and *p*-SMAD2/3 overnight at 4 °C. The sections were then incubated with the indicated secondary antibodies. The percentage of stained positive areas was quantified by ImageJ software.

In order to further observe the degree of liver fibrosis, immunofluorescence experiments were performed as previously described, with some modifications [10]. Briefly, the liver paraffin sections were dewaxed and rehydrated with gradient xylene/alcohol and PBS. The objective tissues were covered with 5% BSA at RT for 30 min and slides were incubated with the first primary antibody overnight at 4 °C. Afterward, they were washed three times with PBS, and slides were then incubated with secondary antibody at RT for 50 min in dark conditions. The procedure was repeated twice with the second and the third set of primary and secondary antibodies. In order to remove the primary antibodies and secondary antibodies combined with tissue, microwave treatments were conducted between each set of antibodies. Afterward, nuclei were stained DAPI for 10 min. After being washed in PBS three times to remove the remaining DAPI, the slides were observed using fluorescence microscopy (Olympus, Tokyo, Japan). The percentage of positive areas was quantified by ImageJ software. Detailed information regarding the antibodies used in IHC and the immunofluorescence experiment is presented in Table S2. 

### 2.8. Bioinformatics Analysis of miRNA Targets

Two types of bioinformatic analysis software, miRanda [16] and RNAhybrid [17], were applied to predict the mRNA targets of sja-let-7 in Mus musculus. The mRNAs were based off the dataset of M. musculus whole transcriptome (GenBank accession no. PRJNA20689). The miRNA potential targeted genes predicted by both software programs were selected for the following gene ontology (GO) analysis [18] and Kyoto Encyclopedia of Genes (KEGG) analysis [19]. The analyses were performed using Metascape, which contains other databases, including Reactome gene sets, WikiPathways, and Comprehensive resource of mammalian protein complexes (CORUM) [20]. The top network clusters were produced by selecting terms based on *p*-value, enrichment factor, and minimum count thresholds. Protein–protein interaction (PPI) enrichment analysis was also performed via the Metascape platform. Proteins that displayed physical interactions with at least one other member of the list according to the default analysis parameters were used for analysis. The Molecular complex detection (MCODE) algorithm was applied using best-scoring by *p*-value threshold to identify densely connected network components. The grouped terms and the PPI network were visualized by Cytoscape (verson 3.10.1) [21].

### 2.9. Dual-Luciferase Reporter Assay

The dual-luciferase reporter assay was conducted according our unpublished manuscript. Briefly, to confirm whether Col1α2 (GenBank accession no. NM_007743.3) was the target gene of sja-let-7, wild-type, or mutant 3′ untranslated regions (UTRs) of Col1α2, the genes were synthesized (GenePharma, China) and then cloned into the pmirGLO luciferase plasmid (Promega, USA). The HEK293T cells (Boster, Wuhan, China) were seeded in a 24-well plate (3 × 10^5^ cells/well). When the cells’ density reached up to 70%, 25 pmol sja-let-7 or NC mimics (GenePharma, Shanghai, China), together with 500 ng wild-type Col1α2 3′UTR plasmid or mutant Col1α2 3′UTR plasmid, were transfected into the HEK293T cells using Lipofectamine 3000 (Thermo Scientific, Waltham, USA). Subsequently, the cells were cultured for 48 h and then collected. A Dual-luciferase Reporter Assay Kit (Promega, Wisconsin, USA) was used to detect the effect of sja-let-7 on the luciferase activity of the Col1α2 3′UTR plasmid. The detailed sequences of miRNA mimics are shown in Table S1.

### 2.10. Fluorescence In Situ Hybridization (FISH)

To demonstrate the relationship between Col1α2 and sja-let-7 on liver sections, FISH was performed. Briefly, according to the tissue fixation time, the slices were boiled in the retrieval solution for 10–15 min and naturally cooled. A total of 20 μg/mL proteinase K (Servicebio, Wuhan, China) was added to the working solution to cover objectives, and the mixture was incubated at 37 °C for 15 min. It was washed in pure water, then washed three times in PBS on a rocker device for, 5 min each. Pre-hybridization solution was added to each section and each section was then incubated for 1 h at 37 °C. Then, the pre-hybridization solution was removed, the sja-let-7 probe hybridization solution with concentration of 500 Nm was added, and the section was incubated in a humidity chamber and hybridized overnight at 40 °C. Then, the hybridization solution was removed. The sections were washed in 2 × SSC (Servicebio, Wuhan, China) for 10 min at 37 °C, in 1 × SSC two times for 5 min each at 37 °C, and in 0.5 × SSC for 10 min at RT. The solution was then discarded, the Col1α2 probe hybridization solution with a concentration of 500 nM was added, and the section was incubated in a humidity chamber and hybridized overnight at 40 °C. The hybridization solution was then removed. Sections were washed with 2 × SSC, 1 × SSC, 0.5 × SSC for 5 min each at 37 °C, respectively. Afterward, nuclei were stained DAPI for 10 min. The slides were observed using a fluorescence microscopy (Olympus, Tokyo, Japan). Detailed information regarding the probes used in the FISH experiment is presented in Table S3.

### 2.11. RNA Extraction and mRNA/miRNA Quantification

To evaluate the level of mRNAs and miRNAs in liver tissues, total RNA was extracted from liver tissues using TRIzol reagent (Invitrogen, Waltham, USA) according to the manufacturer’s instructions [22] and quantified by Nanodrop (Thermo Scientific, Waltham, USA). For mRNAs, reverse-transcription was performed using a Hifair^®^ Ⅲ 1st Strand cDNA Synthesis SuperMix for qPCR kit (Yeasen, Shanghai, China). The resulting cDNA was used as the template for qPCR with Hieff^®^ qPCR SYBR Green Master Mix (Yeasen, Shanghai, China). The relative mRNA expression levels of the genes were quantified with Gapdh, which served as an endogenous control. The LightCycler 96 system (Roche, Shanghai, China) was used for qPCR analysis. The cycling conditions were as follows: preincubation, 95 °C for 60 s; 2-step amplification, 95 °C for 5 s, and 60 °C for 30 s, for 40 cycles; melting, 95 °C for 10 s, 65 °C for 60 s, 97 °C for 1 s. For miRNAs, the first-strand cDNA was reverse-transcribed using the miRNA First Strand cDNA Synthesis kit (Stem-loop Method) (Sangon biotech, Shanghai, China) with a stem-loop RT primer designed by each miRNA. The resulting miRNA cDNA was used as template for qPCR with a MicroRNAs qPCR Kit (SYBR Green Method) (Sangon biotech, Shanghai, China). The relative expression levels of the miRNAs were quantified with U6, which served as an endogenous control. The LightCycler 96 system (Roche, Shanghai, China) was used for qPCR analysis. The cycling conditions were as follows: preincubation, 95 °C for 60 s; 2-step amplification, 95 °C for 5 s, and 62 °C for 30 s, for 40 cycles; melting, 95 °C for 10 s, 65 °C for 60 s, 97 °C for 1 s. All samples were assessed in triplicate.

To evaluate the level of miRNAs in the serum, all miRNAs in serum from BALB/c mice were extracted using a miRcute Serum/Plasma miRNA Isolation Kit (TIANGEN, Shanghai, China) according to the manufacturer’s recommendations. The procedures for first-strand cDNA reverse-transcription and qPCR for miRNAs were the same as detailed above.

The 2^−ΔΔCt^ method [23] was used to calculate the fold change in the expression of all the mRNAs and miRNAs and all samples were assessed in triplicate. The primers used in this study are listed in Table S4.

### 2.12. Establishment of Schistosome-Induced Liver Fibrosis Mice Model

*S. japonicum* cercariae were obtained from the National Reference Laboratory for Animal Schistosomiasis, Shanghai Veterinary Research Institute. A total of 18 BALB/c mice which were percutaneously infected with 20 ± 2 cercariae for the establishment of the liver fibrosis model were divided into *Sj*, *Sj* + NC agomir and *Sj*+Sja-let-7 agomir groups (*n* = 6). In the *Sj* + NC agomir and *Sj*+Sja-let-7 agomir groups, mice were injected with 120 μL of 1 OD NC or Sja-let-7 agomir via the tail vein once a week for six weeks, respectively. In another normal saline (control) group, mice were uninfected with cercariae and were injected with 120 μL PBS via the tail vein once a week for six weeks (*n* = 6). All mice were sacrificed at 6 weeks post infection, and liver tissues from each mouse were collected for histological analysis. 

### 2.13. Statistical Analysis

Data were analyzed with SPSS 25.0 software (SPSS Inc.,New York, USA) and expressed as the mean ± standard deviation (SD) of three independent biological replicates. Data were statistically analyzed with Student’s *t*-tests. A *p*-value of <0.05 was considered statistically significant during statistical analysis.

## 3. Results

### 3.1. Sja-Let-7 Attenuated Progression of CCL_4_-Induced LF in Mice

**Figure 1:**
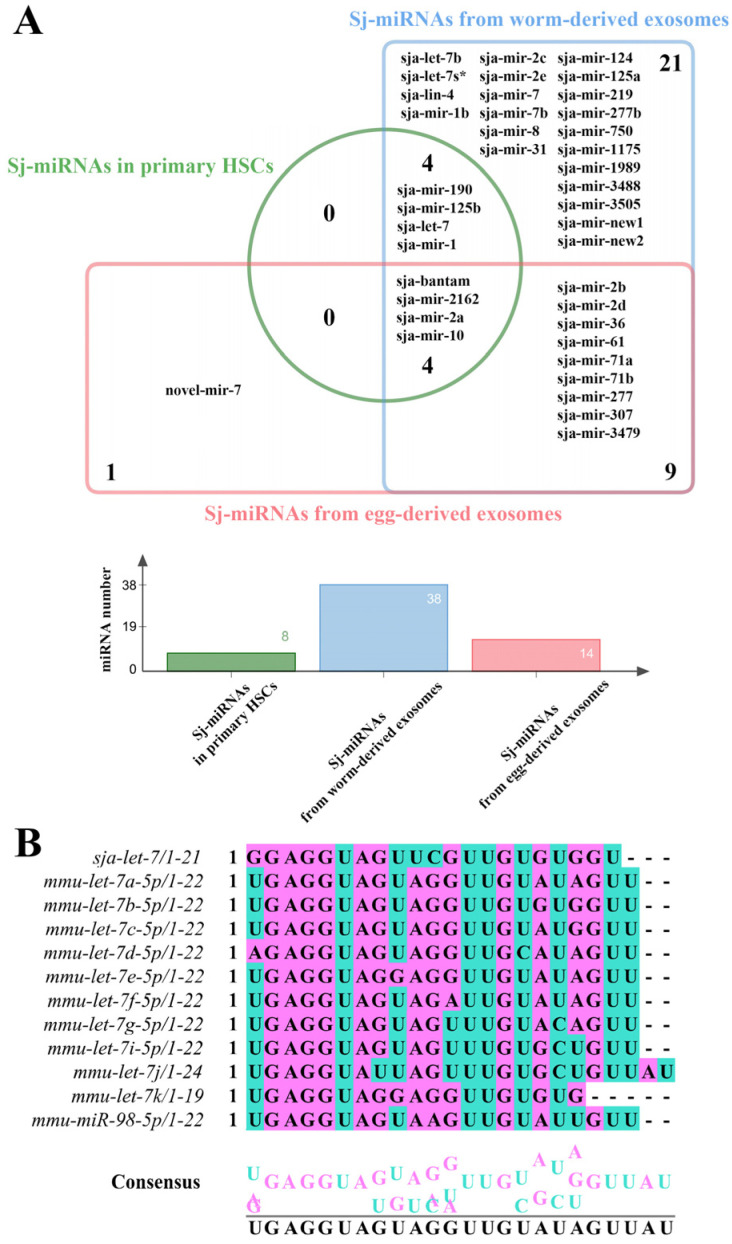
Origin of sja-let-7 and its mammalian family members. (**A**). Compare of *Sj*-miRNAs coming from *S. japonicum* worm-derived, egg-derived EVs to primary HSCs of infected mice; (**B**). Alignments of multiple let-7 mammalian family members.

**Figure 2:**
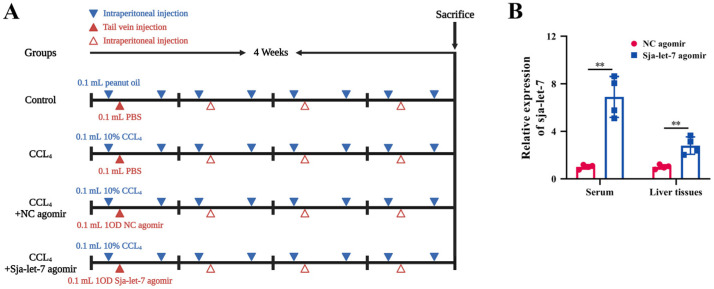
Construction and evaluation of CCL_4_-induced LF model. (**A**) Establishment timeline of CCL_4_-induced LF in a mouse model. (**B**) Detection of sja-let-7 expression in the liver and serum. Each individual is represented by one dot. All graph data are expressed as the mean ± SD of at least three biological replicates per group. Data were statistically analyzed with Student’s *t*-tests. ** *p* < 0.01.

**Figure 3:**
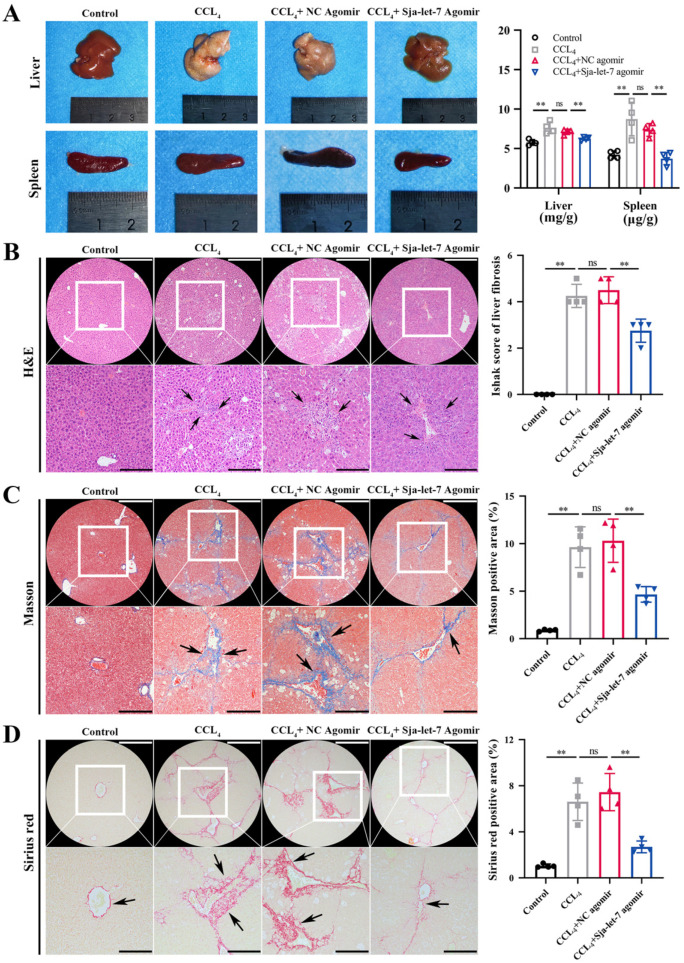
Sja-let-7 attenuates clinical activity of CCL_4_-induced LF mice. (**A**) Liver and spleen appearance and indexes. (**B**) Liver histological analysis and Ishak score of liver fibrosis with H&E staining. (**C**) Liver histological analysis and positive area calculation with Masson staining. (**D**) Liver histological analysis and positive area calculation with Sirius red staining. Black arrows indicate the positive staining area. Scale bar, 200 μm. Insets show a higher magnification of the outlined area. Scale bar, 100 μm. Each individual is represented by one dot. All graph data are expressed as the mean ± SD of at least three biological replicates per group. Data were statistically analyzed with Student’s *t*-tests. ** *p* < 0.01, ns, not significant.

The identification of sja-let-7 in worm-derived EVs and the primary HSCs of infected hosts suggests a potential link to the activation of HSCs (Figure 1A and Table S5) [9]. Based on the same seed sequence and highly conserved functions across species (Figure 1B), an agomir of sja-let-7 was synthesized and applied in a mouse model of CCL_4_-induced LF (Figure 2A). Analysis of serum samples and liver tissue specimens from mice injected with the sja-let-7 agomir for 4 weeks revealed significant up-regulation of miRNAs (Figure 2B), suggesting that sja-let-7 was processed and matured. Administration of the sja-let-7 agomir markedly ameliorated hepatosplenomegaly (Figure 3A) and reduced the liver content of hydroxyproline (Figure S1A). Interestingly, there was no significant change to the hematological index or AST and ALT levels of the mice treated with the sja-let-7 agomir, indicating that injection of CCL_4_ the day before sampling caused acute liver injury (Figure S1B,C). Additionally, hematoxylin and eosin (H&E) staining of liver sections showed that the Ishak score of LF was significantly reduced in the sja-let-7 agomir group as compared to the control group (Figure 3B). Furthermore, Masson and Sirius red staining of liver sections showed significantly reduced collagen deposition in the sja-let-7 agomir-treated mice (Figure 3C,D). Collectively, these results suggest that sja-let-7 attenuated CCL_4_-induced LF.

### 3.2. Sja-Let-7 Diminished Pro-Fibrotic and Pro-Inflammatory Cytokines in CCL_4_-Induced LF

**Figure 4:**
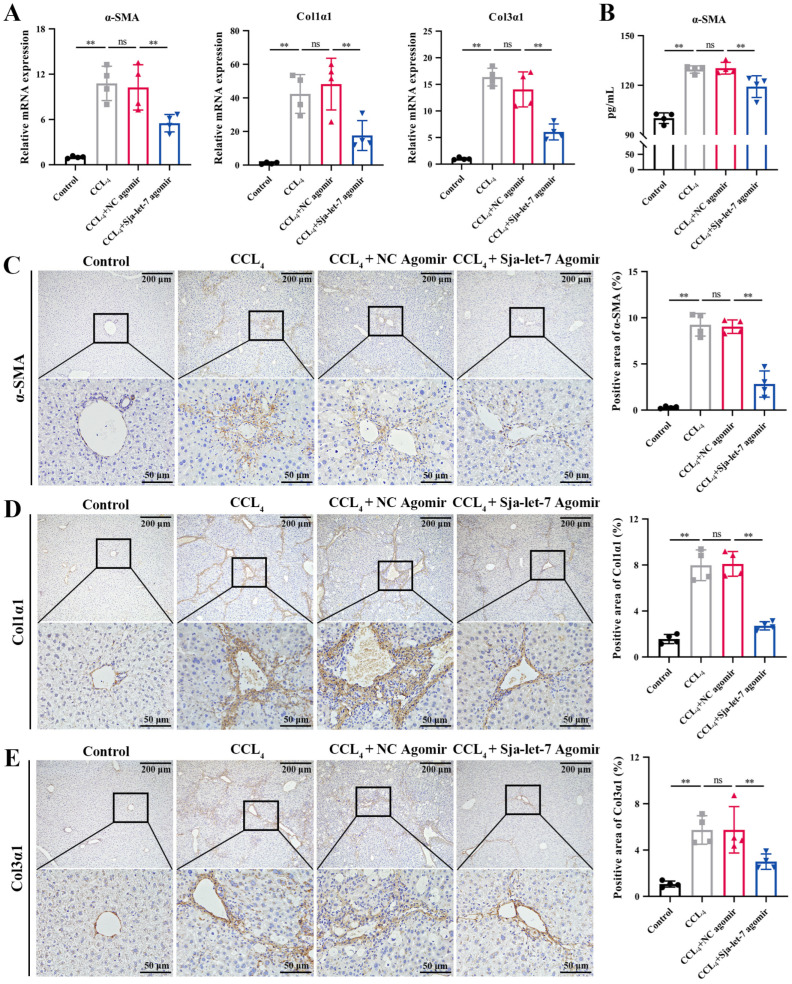
Sja-let-7 diminished pro-fibrotic markers and pro-inflammatory cytokines in CCL_4_-induced LF. (**A**) Detection of α-SMA, Col1α1 and Col3α1 relative expression in the liver. (**B**) ELISA of circulating α-SMA level. (**C**) Liver IHC analysis and positive area calculation of α-SMA. Scale bar, 200 μm. Insets show a higher magnification of the outlined area. Scale bar, 50 μm. (**D**) Liver IHC analysis and positive area calculation of Col1α1. Scale bar, 200 μm. Insets show a higher magnification of the outlined area. Scale bar, 50 μm. (**E**) Liver IHC analysis and positive area calculation of Col3α1. Scale bar, 200 μm. Insets show a higher magnification of the outlined area. Scale bar, 50 μm. Each individual is represented by one dot. All graph data are expressed as the mean ± SD of at least three biological replicates per group. Data were statistically analyzed with Student’s *t*-tests. ** *p* < 0.01, ns, not significant.

**Figure 5:**
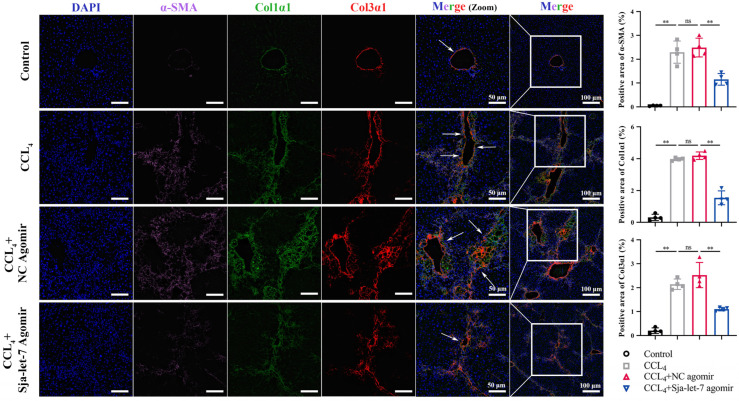
Immunofluorescence analysis and positive area calculation of 3 fibrotic markers after treated with sja-let-7 agomir. White arrows indicate the collagen deposition. Scale bar, 100 μm. Insets show a higher magnification of the outlined area. Scale bar, 50 μm. Each individual is represented by one dot. All graph data are expressed as the mean ± SD of at least three biological replicates per group. Data were statistically analyzed with Student’s *t*-tests. ** *p* < 0.01, ns, not significant.

Next, qPCR analysis of the liver tissues of mice from all groups was conducted to determine the effects of sja-let-7 on pro-fibrotic markers. The results showed that the mRNA levels of three markers of LF (α-SMA, Col1α1, and Col3α1) were significantly reduced (Figure 4A). In addition, the ELISA results revealed that the serum levels of α-SMA were significantly decreased in the sja-let-7 agomir group (Figure 4B). Meanwhile, the results of IHC and immunofluorescence analyses showed significant reductions in the expression levels of all three markers of LF in the sja-let-7 agomir group as compared to the CCL_4_ group (Figure 4C–E and Figure 5).

The process of LF is associated with the secretion of large amounts of inflammatory cytokines. Therefore, several inflammatory cytokines, including IL-1β, IL-6, TNF-α, and HMGB1, were selected for qPCR analysis. The results revealed that the expression levels of all four cytokines were significantly reduced in the sja-let-7 agomir-treated mice (Figure S2). Taken together, these findings indicate that sja-let-7 attenuated progression of CCL_4_-induced LF and ameliorated liver inflammation in vivo.

### 3.3. Sja-Let-7 Regulates LF via Targeting Col1α2

**Figure 6:**
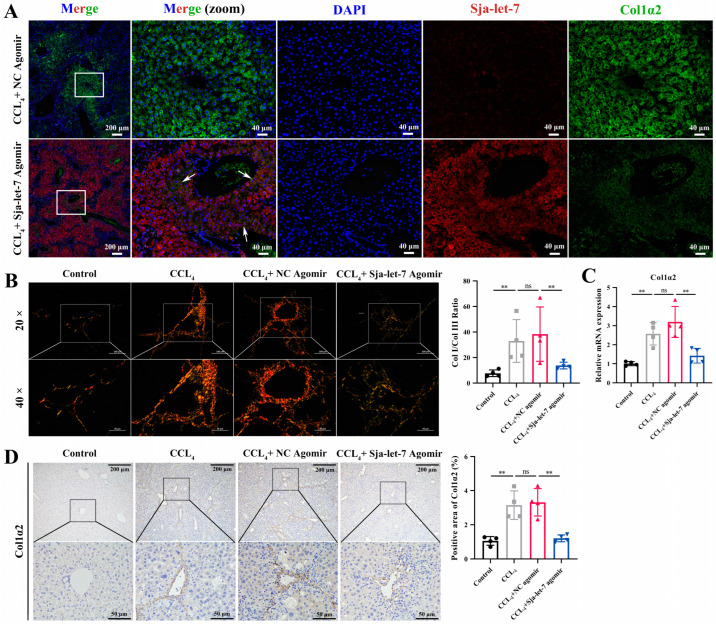
Sja-let-7 regulates LF via targeting Col1α2. (**A**) FISH analysis on the liver section. Scale bar, 200 μm. Insets show a higher magnification of the outlined area. Scale bar, 40 μm. White arrows indicate the cells that co-located with sja-let-7 and Col1α2. (**B**) Polarization microscopy observation of type I and type III collagen fibers and ratio calculation. Scale bar, 100 μm. Insets show a higher magnification of the outlined area. Scale bar, 50 μm. (**C**) Detection of Col1α2 relative mRNA expression in the liver. (**D**) Liver IHC analysis and positive area calculation of Col1α2. Scale bar, 200 μm. Insets show a higher magnification of the outlined area. Scale bar, 50 μm. Each individual is represented by one dot. All graph data are expressed as the mean ± SD of at least three biological replicates per group. Data were statistically analyzed with Student’s *t*-tests. ** *p* < 0.01, ns, not significant.

To further explore the mechanism of sja-let-7 in alleviating LF, 1167 potential target genes that had been predicted with the miRanda algorithm to identify genomic targets of miRNAs [16] and the RNAhybrid tool to determine the minimum free energy hybridization of a long and a short RNA [17] were selected for pathway and process enrichment analyses (Figure S3A and Table S6). As shown in Figure S4B, the two most enriched annotations of the potential target genes were “regulation of system process” and “cell activation”, which were closely related to “regulation of transmembrane transport” and “regulation of exocytosis”, suggesting possible involvement with EV transport (Figure S3C). Interestingly, the PPI network and MCODE algorithm revealed that 10 genes were associated with the term “collagen chain trimerization”, with the highest number of genes (*n* = 4) potentially interacting with Col1α2 (Figure S3D). Therefore, as an important component of type I collagen, Col1α2 was selected for further analysis. A luciferase reporter plasmid was then constructed based on the sja-let-7 binding site of the 3′-untranslated region of *Mus musculus* Col1α2. The results of the dual-luciferase reporter assays showed that the sja-let-7 mimic significantly reduced the luciferase activity of the Col1α2 constructs as compared to the NC mimic (Figure S3E). To further verify the correlation between Col1α2 and sja-let-7, which was overexpressed by miRNA agomir in vivo, FISH analysis of mouse liver slices was conducted. As shown in Figure 6A, after overexpression of sja-let-7, the expression of which was widely presented in whole liver while the expression of its target gene Col1α2 was relatively low around the collagen fiber deposits, they were found to still be co-localized with the nuclei, thereby confirming a targeting relationship. Comparatively, there was no obvious sja-let-7 expression or co-localization in the NC group.

To determine whether sja-let-7 regulates CCL_4_-induced LF via Col1α2, type I and III collagen fibers were quantified by polarization microscopy. As shown in Figure 6B, the amount of type I collagen fibers, which were stained bright red or yellow, was significantly reduced in the liver tissues of mice treated with the sja-let-7 agomir. Moreover, the results of qPCR and IHC analyses showed that overexpression of sja-let-7 significantly reduced the mRNA and protein levels of Col1α2 (Figure 6C, D).

### 3.4. Sja-Let-7 Regulates LF via the TGF-β/Smad Pathway

**Figure 7:**
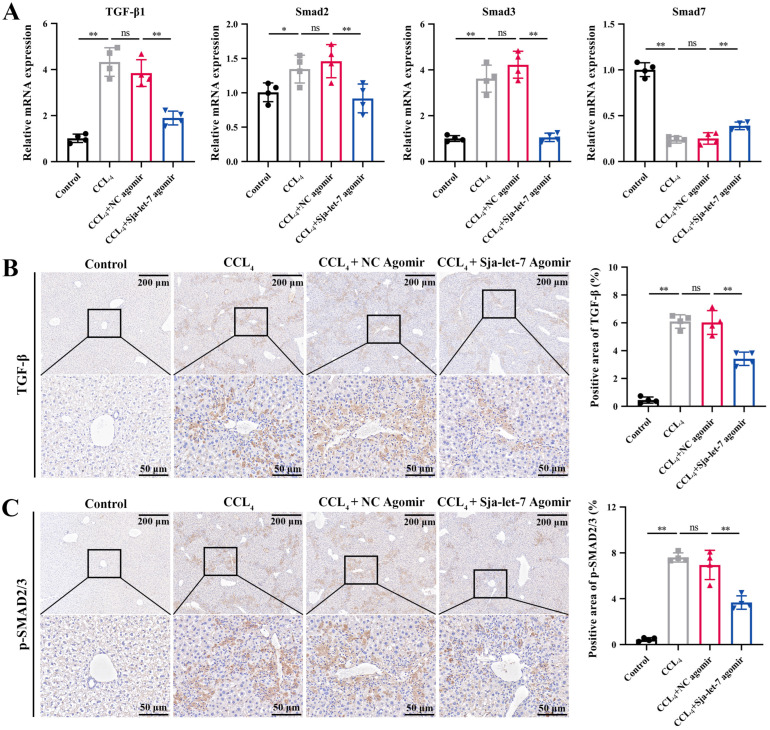
Sja-let-7 regulates LF through TGF-β/Smad pathway. (**A**) Detection of TGF-β, Smad2, Smad3, and Smad7 relative expression in the liver. (**B**) Liver IHC analysis and positive area calculation of TGF-β. Scale bar, 200 μm. Insets show a higher magnification of the outlined area. Scale bar, 50 μm. (**C**) Liver IHC analysis and positive area calculation of *p*-smad2/3. Scale bar, 200 μm. Insets show a higher magnification of the outlined area. Scale bar, 50 μm. Each individual is represented by one dot. All graph data are expressed as the mean ± SD of at least three biological replicates per group. Data were statistically analyzed with Student’s *t*-tests.* *p* < 0.05, ** *p* < 0.01, ns, not significant.

Pathway and process enrichment analyses showed that the target gene of sja-let-7 was enriched in the term “SMAD2/SMAD3:SMAD4 heterotrimer regulates transcription” (Figure S4), indicating that Col1α2, as an important component of type I collagen, might be regulated by the fibrotic TGF-β/Smad pathway. Therefore, qPCR and IHC analyses were conducted to determine the effects of sja-let-7 overexpression on molecules downstream of the TGF-β/Smad signaling pathway. The results revealed that treatment with the sja-let-7 agomir significantly reduced the mRNA and protein levels of Smad2 and Smad3, as key promoters of the TGF-β/Smad signaling pathway, while expression of the antagonist Smad7 was increased (Figure 7A–C). Collectively, these results demonstrate that sja-let-7 inhibited CCL_4_-induced LF via the TGF-β/Smad signaling pathway.

### 3.5. Sja-Let-7 Reduces Schistosome-Induced LF

**Figure 8:**
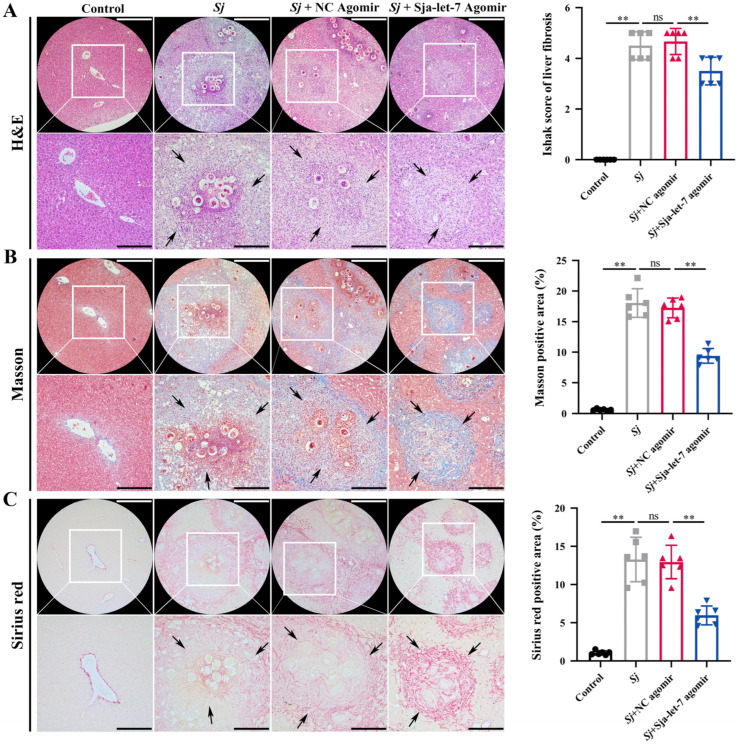
Sja-let-7 reduces schistosome-induced LF. (**A**) Liver histological analysis and Ishak score of liver fibrosis with H&E staining. (**B**) Liver histological analysis and positive area calculation with Masson staining. (**C**) Liver histological analysis and positive area calculation with Sirius red staining. Black arrows indicate the egg granuloma. Scale bar, 200 μm. Insets show a higher magnification of the outlined area. Scale bar, 100 μm. Each individual is represented by one dot. All graph data are expressed as the mean ± SD of at least three biological replicates per group. Data were statistically analyzed with Student’s *t*-tests. ** *p* < 0.01, ns, not significant.

Histological analysis of mouse liver tissues was conducted to determine whether the anti-fibrotic effect of sja-let-7 also reduces schistosome-induced LF. H&E, Masson, and Sirius red staining showed significantly reduced collagen deposition in the liver sections of mice treated with the sja-let-7 agomir (Figure 8).

## 4. Discussion

**Figure 9:**
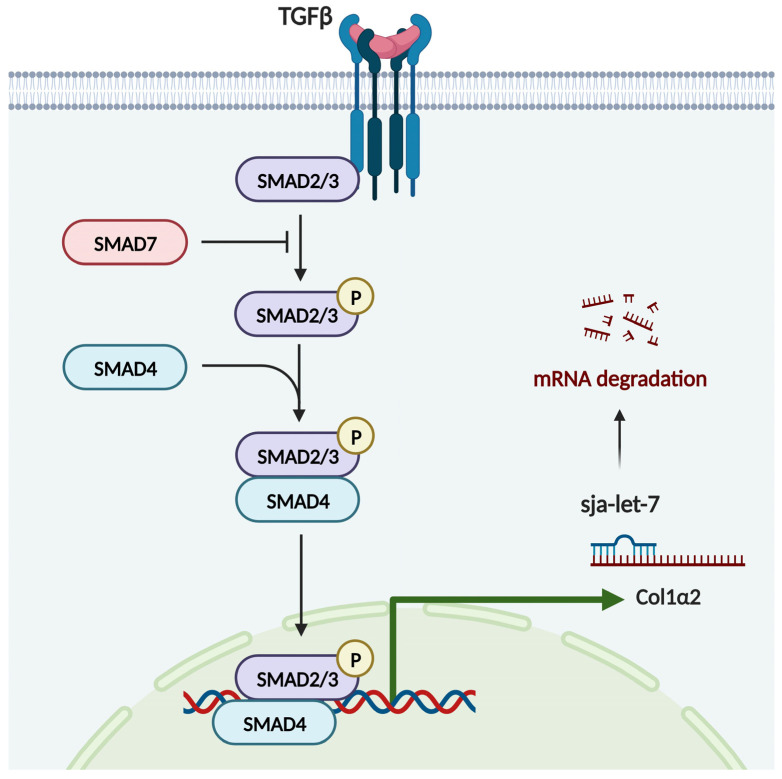
Graphical abstract of the mechanism. Sja-let-7 suppress the CCL_4_-induced LF via Col1α2 and TGF-β/Smad signaling pathway. This figure was created by Biorender.com.

In the present study, the effect of sja-let-7 on CCL_4_-induced LF was assessed. As expected, sja-let-7 attenuated CCL_4_-induced LF in mice. Specifically, sja-let-7 reduced the expression of pro-fibrotic markers and pro-inflammatory cytokines in CCL_4_-induced LF via the TGF-β/Smad signaling pathway (Figure 9).

Various schistosome-derived molecules have been evaluated for the treatment of immune and inflammatory diseases based on observations that schistosomes modulate the host immune response, as described by the “hygiene hypothesis” and “old friends hypothesis”. For example, a recombinant 16-kDa protein secreted by *S. japonicum* was shown to attenuate dextran sulfate sodium-induced colitis in mice, which was attributed to a reduced production of pro-inflammatory cytokines, an increased differentiation of regulatory T cells, and the inhibition of the PPAR-α signaling pathway [24]. Likewise, glutathione S-transferase of *S. haematobium* was reported to modulate immune responses in a mouse model of colitis [25]. Interestingly, the schistosome-derived molecules sja-miR-71a [26] and sja-miR-3096 [27] exhibited anti-tumor effects through the modulation of tumor-related genes. 

The roles ofEVs as important carriers of schistosome-derived miRNAs to host cells have received extensive attention. Sja-miR-125b and Sja-bantam transported by EVs of *S. japonicum* were shown to promote the proliferation of macrophages and upregulate the expression of TNF-α, thereby modulating the host immune response [28]. Sma-miR-10 transported by EVs of *S. mansoni* targets MAP3K7 and consequently downregulates NF-κB activity by mediating the differentiation of Th2 cells [29]. In addition, tpi-let-7 enriched in exosome-like vesicles from *Taenia pisiformis*, a parasitic tapeworm of wild rabbits, induced M2 macrophage polarization by targeting CCAAT/enhancer-binding protein-δ [30]. Together, the results of these studies demonstrate the therapeutic potential of helminth-derived miRNAs, thus warranting further investigations.

Reviews of several helminth EVs databases [28,31,32] found relatively high expression of let-7 family members, which are negatively correlated with fibrosis in many organs of mammals as potential targets for immunotherapy [33,34,35]. However, the relationship between helminth-derived let-7 and LF remains unclear. A previous study [9] found that sja-let-7 of *S. japonicum* in primary HSCs plays an important regulatory role in a mouse model of LF (Figure 1A). A prior investigation by our group confirmed that sja-let-7 attenuated *S. japonicum*-induced LF. Although these findings were not published, the experiments were repeated in the present study with a different animal cohort. Through liver histological observation, the Ishak score of liver fibrosis, Masson, and Sirius red positive areas were significantly reduced, which demonstrated the similar roles of sja-let-7 in the etiology of LF.

CCL_4_ generates free CCL^3−^ and CL^−^, which can covalently bind to the macromolecules in hepatocytes, initiating lipid peroxidation and the production of reactive oxygen species, resulting in hepatocellular necrosis, chronic inflammation, and LF [36]. To test this hypothesis, a mouse model of CCL_4_-induced LF was constructed, which shares similarities with schistosome-induced LF, especially the involvement of inflammatory cytokines, macrophages, and T lymphocytes [37]. The liver histological observation showed that the overexpression of sja-let-7 significantly inhibited the expression of fibrotic marker α-SMA, Col1α1, and Col3α1, which indicated that the progression of CCL_4_-induced LF was reduced. In addition, the mRNA expression levels of IL-1β, IL-6, and TNF-α, which are involved in both CCL_4_-induced and schistosome-induced LF [37], were also significantly reduced after treatment with the sja-let-7 agomir, indicating the amelioration of liver inflammation.

In terms of mechanisms, bioinformatics analysis as well as previous studies of schistosome-induced LF by our group (unpublished manuscript) confirmed that the TGF-β/Smad signaling pathway is involved in the regulation of LF. In addition, the results of the present study verified that sja-let-7 targets Col1α2 and that the activation of this signaling pathway was inhibited by overexpression of sja-let-7, in agreement with the model of schistosome-induced LF.

This study is the first to apply worm EV-derived miRNAs to a mouse model of CCL_4_-induced LF, thereby providing an important reference for future applications of worm-derived immunomodulatory molecules. However, since a mouse model was employed in this study, the generality of the outcomes is limited. In addition, further studies are needed to determine whether worm-derived molecules cause side effects to the host.

## 5. Conclusions

In conclusion, sja-let-7 derived from *S. japonicum* worms was overexpressed in a mouse model of CCL_4_-induced LF, which could target Col1α2 and the TGF-β/Smad signaling pathway to attenuate the progression of LF.

## Data Availability

The original contributions presented in the study are included in the article. Further inquiries can be directed to the corresponding authors.

## Supplementary Materials

The following supporting information can be downloaded at: https://www.mdpi.com/article/10.3390/biology12121465/s1, Figure S1: Hydroxyproline, AST and ALT content in the liver and hematological index from mice treated with sja-let-7 agomir. A. Hydroxyproline content; B. AST and ALT content; C. Hematological index. Each individual is represented by one dot. All graph data are expressed as the mean ± SD of at least three biological replicates per group. ** *p* < 0.01, ns, not significant. AST—aspartate aminotransferase, ALT—aminotransferase, WBC—White blood cells, Neu—neutrophil, Lym—lymphocyte, Mon—monocyte, Eos—eosinophil and Bas—basophil. Figure S2: mRNA expression of pro-inflammatory cytokines TNF-α, IL-1β, IL-6, and HMGB1. Each individual is represented by one dot. All graph data are expressed as the mean ± SD of at least three biological replicates per group. ** *p* < 0.01, ns, not significant. Figure S3: Col1α2/TGF-β/Smad axis is the targeting pathway of sja-let-7. A. Venn diagram showing 1167 potential target genes overlap in miRanda and RNAhybrid database; B. Bar graph of enriched terms; C. PPI networks identified in 1167 potential target genes; D. and MCODE component related to Col1α2; E. The dual-luciferase reporter assay. Each individual is represented by one dot. All graph data are expressed as the mean ± SD of at least three biological replicates per group. ** *p* < 0.01, ns, not significant. WT: wild type, MUT: mutant. Figure S4: Top 55 enriched terms related to 1167 target genes of sja-let-7. Table S1: The sequence of miRNA mimics, inhibitor, and agomir. Table S2: Antibodies used in the experiment. Table S3: Probes used in the FISH analysis. Table S4: Primers used in the experiment. Table S5: Comparison of Sj-miRNAs coming from *S. japonicum* worm-derived, egg-derived EVs s to primary HSCs of infected mice. Table S6: Potential target genes of sja-let-7 identified by both RNAhybrid and miRanda.
