# Supplementary material for: Sja-Let-7 Attenuates Carbon Tetrachloride-Induced Liver Fibrosis in a Mouse Model via Col1α2
Source: Biology (Basel). 2023 Nov 24;12(12):1465. doi: 10.3390/biology12121465 (PMC10740823; doi:10.3390/biology12121465)
Supplement: Supplementary file 1 [file biology-12-01465-s001.zip › Supporting materials.pdf]

Contents:

1. Supplementary figures and figure legends

Supplementary Figure S1

Supplementary Figure S2

Supplementary Figure S3

Supplementary Figure S4

2. Supplementary tables

Supplementary Table S1

Supplementary Table S2

Supplementary Table S3

Supplementary Table S4

Supplementary figures and figure legends

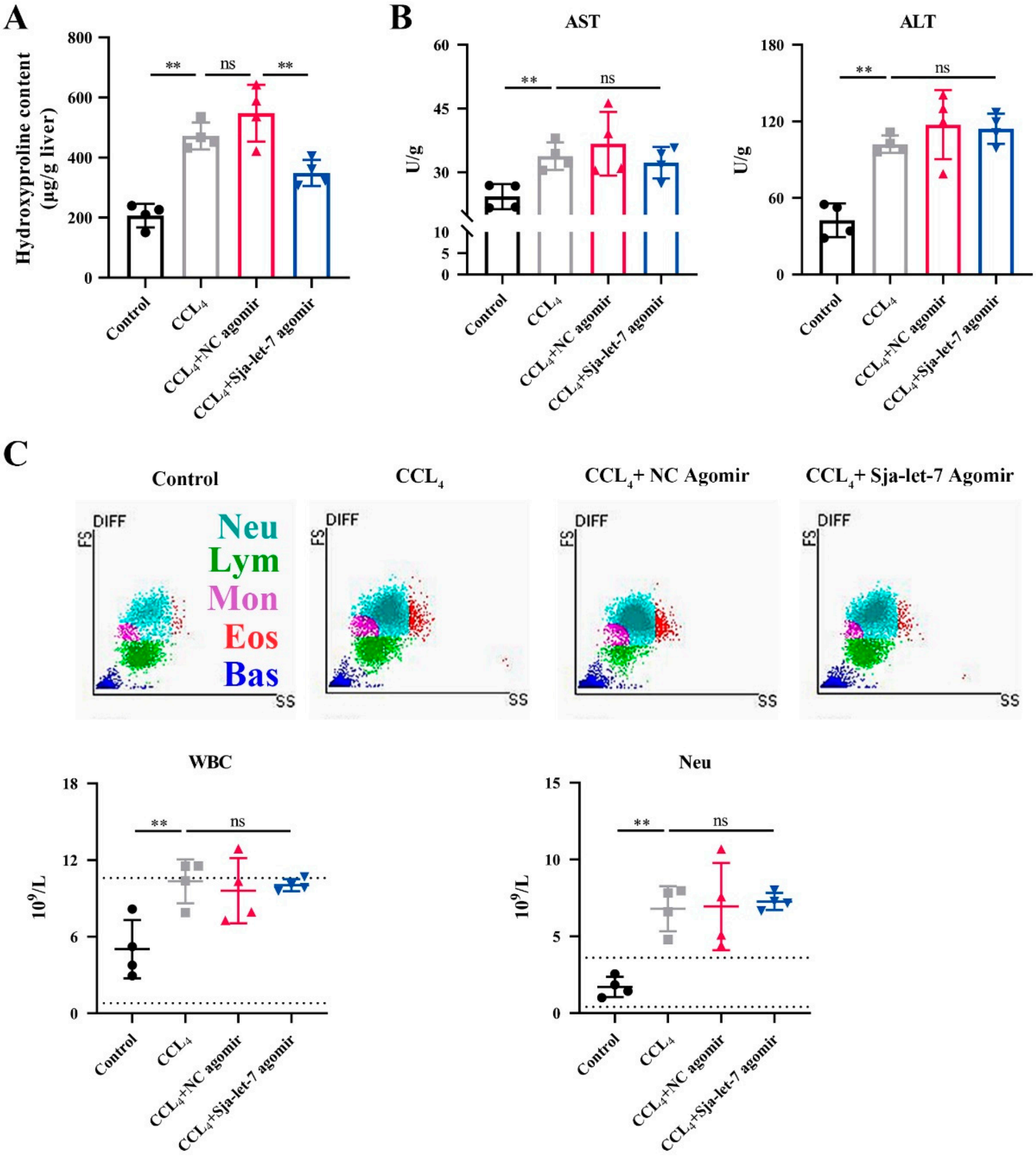

Supplementary Figure S1. Hydroxyproline, AST and ALT content in the liver and hematological index from mice treated with sja-let-7 agomir. A. Hydroxyproline content; **B.** AST and ALT content; **C.** Hematological index. Each individual is represented by one dot. All graph data are expressed as the mean ± SD of at least three biological replicates per group. **p< 0.01, ns, not significant. AST: aspartate aminotransferase, ALT: aminotransferase, WBC: White blood cells, Neu: neutrophil, Lym: lymphocyte, Mon: monocyte, Eos: eosinophil and Bas: basophil.

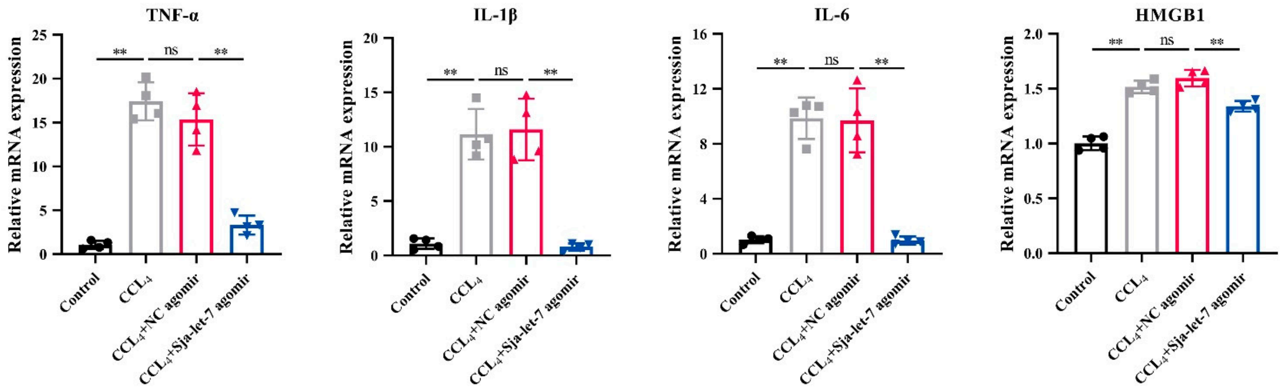

Supplementary Figure S2. mRNA expression of pro-inflammatory cytokines TNF-α, IL-1β, IL-6 and HMGB1. Each individual is represented by one dot. All graph data are expressed as the mean ± SD of at least three biological replicates per group. ***p*< 0.01, ns, not significant.

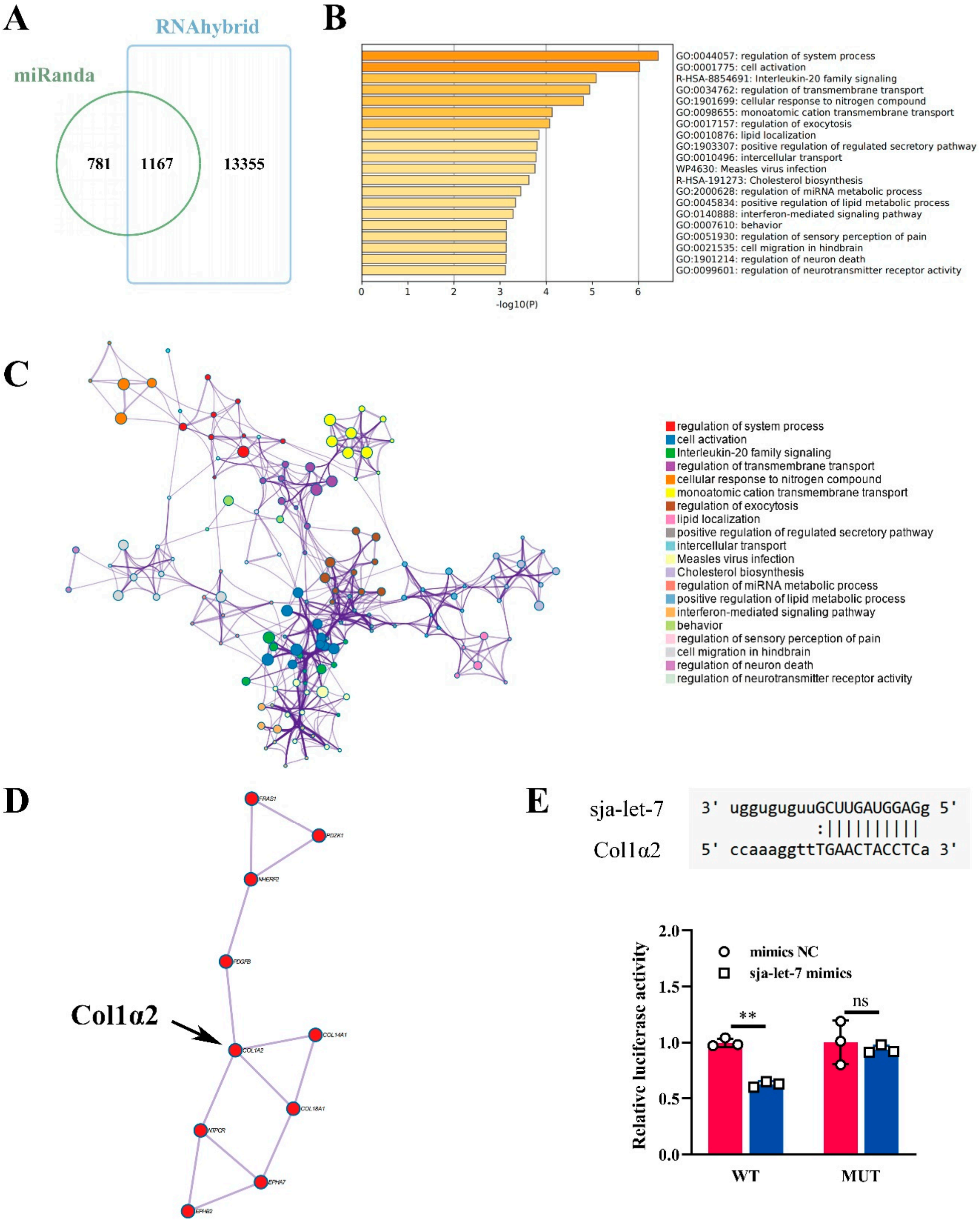

Supplementary Figure S3. Col1α2/TGF-β/Smad axis is the targeting pathway of sja-let-7. A. Venn diagram showing 1167 potential target genes overlap in miRanda and RNAhybrid database; **B.** Bar graph of enriched terms; **C.** PPI networks identified in 1167 potential target genes; **D.** and MCODE component related to Col1α2; **E.** The dual-luciferase reporter assay. Each individual is represented by one dot. All graph data are expressed as the mean ± SD of at least three biological replicates per group. **$p < 0.01$, ns, not significant. WT: wild type, MUT: mutant.

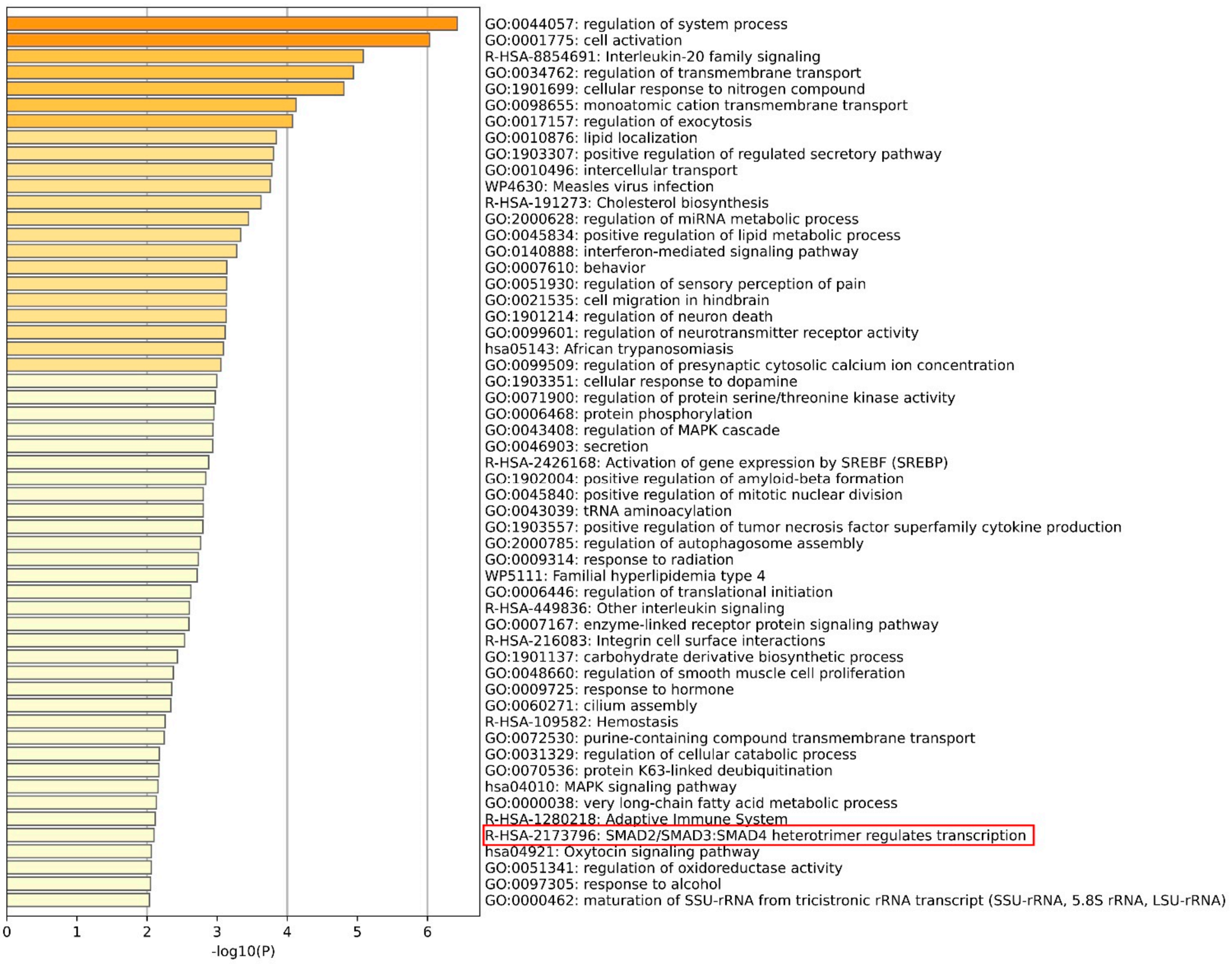

Supplementary Figure S4. Top 55 enriched terms related to 1167 target genes of sja-let-7.

Supplementary tables

Supplementary Table S1 The sequence of miRNA mimics, inhibitor and agomir.

Name	Sense sequence (5'-3')	Anti-sense sequence (5'-3')
Sja-let-7 agomir	GGAGGUAGUUCGUUGUGUGGU	CACACAACGAACUACCUCCUU
NC agomir	UUCUCCGAACGUGUCACGUTT	ACGUGACACGUUCGGAGAATT
Sja-let-7 mimics	GGAGGUAGUUCGUUGUGUGGU	CACACAACGAACUACCUCCUU
NC mimics	UUCUCCGAACGUGUCACGUTT	ACGUGACACGUUCGGAGAATT

Supplementary Table S2 Antibodies used in the experiment

Experiments	Primary antibody	Source (Catalogue No.)	Host	Working conditions	Secondary antibody	Source (Catalogue No.)	Working conditions
**Immunohistochemical assay**	TGF-β1	Servicebio (GB11179)	Rabbit	1:500	HRP conjugated Goat Anti-Rabbit IgG (H+L)	Servicebio (GB23303)	1:200
	p-SMAD2/3	SAB (12241)	Rabbit	1:200			
	α-SMA	Servicebio (GB111364)	Rabbit	1:2000			
	Col1α1	Servicebio (GB11022)	Rabbit	1:1000			
	Col1α2	Proteintech (14695-1-AP)	Rabbit	1:500			
	Col3α1	Servicebio (GB111629)	Rabbit	1:500			
**Immunofluorescence analysis**	α-SMA	Servicebio (GB13044)	Mouse	1:1000	Cy5 conjugated Goat Anti-mouse IgG (H+L)	Servicebio (GB27301)	1:400
	Col1α1	Servicebio (GB11022)	Rabbit	1:3000	Cy3 conjugated Goat Anti-Rabbit IgG (H+L)	Servicebio (GB21303)	1:300
	Col3α1	Servicebio (GB111629)	Rabbit	1:200	Alexa Fluor® 488-conjugated Goat Anti-Rabbit IgG (H+L)	Servicebio (GB25303)	1:400

Supplementary Table S3 Probes used in the FISH analysis.

**Probe name**	**Sequence**
sja-let-7	5’-ACCACACAACGAACTACCTCC-3’
	5’-TGTCTTGCCCCATTCATTTGTCTTTTT-3’
Col1α2	5’-CAGGCGAGATGGCTTATTTGTTTTGT-3’
	5’-GGCATGTTGCTAGGCACGAAGTTACT-3’

Supplementary Table S4 Primers used in the experiment

Primer	Sequences (5'-3')
Mouse-GAPDH-F	AACGGGAAGCCCATCACCATC
Mouse-GAPDH-R	AAGACACCAGTAGACTCCACGA
Mouse-IL-1β-F	ATGAAAGACGGCACACCCAC
Mouse-IL-1β-R	GCTTGTGCTCTGCTTGTGAG
Mouse-IL-6-F	TGCAAGAGACTTCCATCCAGT
Mouse-IL-6-R	GTGAAGTAGGGAAGGCCG
Mouse-TNFα-F	CAGCCGATGGGTTGTACCTT
Mouse-TNFα-R	TGTGGGTGAGGAGCACGTAGT
Mouse-HMGB1-F	GGCGAGCATCCTGGCTTATC
Mouse-HMGB1-R	GGCTGCTTGTCATCTGCTG
Mouse-α-SMA-F	TCAGCGCCTCCAGTTCCT
Mouse-α-SMA-R	AAAAAAAACCACGAGTAACAAATCAA
Mouse-Col1α1-F	ACGTCCTGGTGAAGTTGGTC
Mouse-Col1α1-R	CAGGGAAGCCTCTTTCTCCT
Mouse-Col1α2-F	CCAGGGCTGTTTTCCCATCC
Mouse-Col1α2-R	GCTCTGTGCTTCGTCACCCA
Mouse-Col3α1-F	GCCCACAGCCTTCTACACCT
Mouse-Col3α1-R	GCCAGGGTCACCATTTCTC
Mouse-TGF-β1-F	ATTCCTGGCGTTACCTTGG
Mouse-TGF-β1-R	AGCCCTGTATTCCGTCTCCT
Mouse-Smad2-F	GTGGCATACTGGGAGGAGAA
Mouse-Smad2-R	TTGTTGTCCGAATTGAGCTG
Mouse-Smad3-F	GAGACATTCCACGCTTCACA
Mouse-Smad3-R	GCTGCATTCCGGTTAACATT
Mouse-Smad7-F	GTGTTGCTGTGAATCTTACG

Mouse-Smad7-R	AGAAGAAGTTGGGAATCTGA
common-REVERSE	CAGTGCAGGGTCCGAGGT
mouse U6-RT-primer	GTCGTATCCAGTGCAGGGTCCGAGGTATTCGCACTGGATACGACAAAAAT
mouse U6-FORWARD	GAAGATTTAGCATGGCCCCTGC
sja-let-7-RT-primer	GTCGTATCCAGTGCAGGGTCCGAGGTATTCGCACTGGATACGACACCACA
sja-let-7-FORWARD	ACAACAACGGAGGTAGTTCGT
